# It takes longer than you think: librarian time spent on systematic review tasks*

**DOI:** 10.5195/jmla.2018.323

**Published:** 2018-04-01

**Authors:** Krystal Bullers, Allison M. Howard, Ardis Hanson, William D. Kearns, John J. Orriola, Randall L. Polo, Kristen A. Sakmar

**Affiliations:** Emerging Technologies and Pharmacy Liaison Librarian, Hinks and Elaine Shimberg Health Sciences Library, University of South Florida, Tampa, FL; Research and Education Librarian, Hinks and Elaine Shimberg Health Sciences Library, University of South Florida, Tampa, FL; Assistant Director, Research and Education, Hinks and Elaine Shimberg Health Sciences Library, University of South Florida, Tampa, FL; Research Associate Professor, Department of Rehabilitation and Mental Health Counseling, University of South Florida, Tampa, FL; Research and Education Librarian, Hinks and Elaine Shimberg Health Sciences Library, University of South Florida, Tampa, FL; Research and Education Librarian, Hinks and Elaine Shimberg Health Sciences Library, University of South Florida, Tampa, FL; Assistant Librarian, Research and Education, Hinks and Elaine Shimberg Health Sciences Library, University of South Florida, Tampa, FL

## Abstract

**Introduction:**

The authors examined the time that medical librarians spent on specific tasks for systematic reviews (SRs): interview process, search strategy development, search strategy translation, documentation, deliverables, search methodology writing, and instruction. We also investigated relationships among the time spent on SR tasks, years of experience, and number of completed SRs to gain a better understanding of the time spent on SR tasks from time, staffing, and project management perspectives.

**Methods:**

A confidential survey and study description were sent to medical library directors who were members of the Association of Academic Health Sciences Libraries as well as librarians serving members of the Association of American Medical Colleges or American Osteopathic Association.

**Results:**

Of the 185 participants, 143 (77%) had worked on an SR within the last 5 years. The number of SRs conducted by participants during their careers ranged from 1 to 500, with a median of 5. The major component of time spent was on search strategy development and translation. Average aggregated time for standard tasks was 26.9 hours, with a median of 18.5 hours. Task time was unrelated to the number of SRs but was positively correlated with years of SR experience.

**Conclusion:**

The time required to conduct the librarian’s discrete tasks in an SR varies substantially, and there are no standard time frames. Librarians with more SR experience spent more time on instruction and interviews; time spent on all other tasks varied widely. Librarians also can expect to spend a significant amount of their time on search strategy development, translation, and writing.

## INTRODUCTION

The National Academy of Medicine, the *Cochrane Handbook for Systematic Reviews of Interventions,* and the Agency for Healthcare Research and Quality each recommend securing the services of a librarian to plan strategically effective and comprehensive searches [1–3]. Recent academic publications have reported increased demand for librarian support services for systematic reviews (SRs) [4–9]. This trend is borne out by Crum and Cooper [10], who identified SR support as the second-most requested role of medical librarians as well as the largest concern for medical library directors in planning for staffing. Librarian-developed SR searches are likely to be thorough and reproducible, as librarians provide better-defined search strategies, use accurate search terminology, and access more databases [11, 12].

SR support from librarians can be as basic as suggesting appropriate databases or possible search terms. However, Knehans et al. have found that librarians often do much more [13]. They have identified the following common librarian tasks: developing and documenting the search strategy, translating the strategy to rerun in other databases, utilizing bibliographic software for the management and removal of duplicates, searching the grey literature, performing hand searches, writing the methods section, and searching through the bibliographies of retrieved articles for additional relevant studies (i.e., pearling).

One of the major variables in performing an SR is the amount of time necessary to conduct the various tasks. Allen and Olkin devised a formula to predict the time required to complete a meta-analysis [14]. Although their time-task analysis was not for an SR, the tasks that they described were very similar to SR tasks and required comparable librarian effort. They estimated it should take from 25 to 2,518 hours, with a mean total of 1,139 hours, to conduct a meta-analysis. Their estimate included 588 hours needed for search, retrieval, and creation of a database for the search results. At the low end of the time spectrum, Saleh et al. concluded that the mean time for completing the SR search would be 24 hours, with a median of less than 8 hours [15]. Gann and Pratt determined the average time for SR search completion was 23 hours, and the average time was 6 hours to update an SR [16]. None of these authors addressed the specific time required for librarians to accomplish the discrete SR tasks. Hence, the extreme variation in times for the overall SR process alludes to the challenges that libraries face in planning for SR support.

Because the strategic plan for the authors’ library anticipated an increase in the demand for SR support services, we sought to gain a fuller understanding of the range in completion times for SR tasks. Due to deficits in the available literature, we developed a survey to discover how much time librarians spent on SR tasks and whether experience was a factor in task duration.

## METHODS

### Design and participants

We developed a mixed methods survey in Qualtrics to query librarians on the amount of time that they spent on their most recent SRs. We hoped to obtain a cross-section of results to provide a good estimate of the lengths of time required, while reducing errors in recollection.

**Figure 1:**
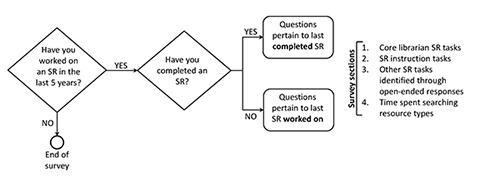
Flowchart of the survey sections

The survey had three unique tracks based on participant response: (1) no SR experience in the past five years, (2) worked on at least one SR in the past five years but completed none, and (3) worked on and completed at least one SR in the past five years. Completion was defined as finishing the respondent’s assigned tasks rather than the overall SR (Figure 1).

The survey contained four sections in each track: (1) core librarian SR tasks, (2) tasks related to SR instruction, (3) other tasks not identified by the survey (open-ended responses), and (4) time spent searching various types of resources. Tasks in sections 1, 2, and 4 were identified and defined based on our professional experience and our review of the literature. For each task, respondents estimated time spent in hours for their most recent SR. The core librarian tasks and definitions used in section 1 were:

- Interview: conducting the initial interview and follow-up meetings
- Search strategy: testing and developing search terms and strategy
- Translation of search: translating search strategy to other databases
- Documentation: documenting search strategy
- Delivery: delivering search results (e.g., bibliographic management tool, shared search statements)
- Writing: writing search methodology section for manuscript (e.g., PRISMA flowcharts)
- Additional tasks: performing any SR-related task not listed

Section 2 was devoted to SR instructional tasks (e.g., SR methodology, literature database management, bibliographic management software, and self-identified “other” instructional tasks). This section explored how much time participants devoted to developing coinvestigator information skills that were necessary to successfully complete an SR. As in section 1, participants estimated time spent in hours.

In section 3, participants volunteered SR-related task information that was not identified by the survey, such as developing the protocol, creating inclusion and exclusion criteria, or analyzing data.

In section 4, participants reported the relative percentage of their time spent in searching the following types of resources: literature databases or indexes, bibliographies of relevant studies (i.e., pearling), trial registers, grey literature, hand searching, and targeted journal titles or conference proceedings (i.e., hand searching).

The survey concluded with an open-ended question allowing participants to reveal other concerns or comments pertaining to librarians’ time spent on SRs. The supplemental appendix provides the full survey.

Prior to distribution, we identified a small group of medical librarians with a range in experience, from expert to novice. They provided a critique of the survey, clarified ambiguous items, and identified content areas requiring elaboration. The University of South Florida’s Research Integrity and Compliance Office reviewed and approved the submitted survey protocol.

The survey was sent via email to: (1) medical library directors subscribing to the Association of Academic Health Sciences Libraries (AAHSL) email discussion list and (2) librarians serving members of the Association of American Medical Colleges (AAMC) and the American Osteopathic Association (AOA). AAHSL recipients were requested to forward the survey to librarians in their institutions who had worked on SRs. Using these lists created a convenience sample, which allowed us to focus the collection of data from other academic health sciences librarians.

The initial solicitation was sent on December 11, 2015. A reminder email to participate was sent on December 17, 2015, and the survey remained open until January 7, 2016. The voluntary solicitation included no incentives. To ensure confidentiality, no names or affiliations were requested.

### Data analysis

All collected data were exported from Qualtrics and analyzed in Microsoft Excel. Additional statistical analyses were conducted using SPSS, version 24. Statistical evaluation proceeded in two phases. Phase 1 consisted of identifying outliers and “bad” data (i.e., incomplete or ambiguous), removing them from the dataset, and generating descriptive statistics for individual items. Phase 2 was an exploratory factor analysis (FA), comprising a principal components analysis (PCA) and a VARIMAX rotation. These were conducted to create a concise model relating the durations of various tasks to the librarian’s experience level. A PCA is an important first step in the search for a unifying factor structure underlying what appears to be disparate measures [17]. Once the PCA was completed, further clarification was achieved by applying a VARIMAX rotation, which maximized differences in the factor structure and clarified relationships among the measures.

## RESULTS

The survey garnered 185 responses; 39 were excluded due to missing data. A further 41 participants who had never participated in an SR in the previous 5 years were excluded, resulting in a total of 105 valid surveys for phase 1 analysis.

The total number of SRs worked on by librarians ranged from 1 to 500, and the distribution was positively skewed, with a median of 5 and a mean of 23.4 (standard deviation [SD]=70.5) SRs. For years of experience, 16 respondents reported less than 1 year, 52 had 1–3 years, 25 had 4–6 years, and 12 had 7 or more years. Table 1 provides detailed results for the time spent in each category of task as a function of the number of SRs completed and the number of years of experience.

**Table 1:** Systematic review (SR) task time spent in hours by experience levels

		Interview		Search strategy		Translate search		Documentation/ delivery/writing		Instruction		Total	

	Count	Average	Median	Average	Median	Average	Median	Average	Median	Average	Median	Average	Median
Number of SRs													
1–2	25	4.3	3.0	6.9	3.0	3.9	3.0	11.1	10.0	4.0	1.5	46.2	36.0
3–5	29	3.6	2.0	9.9	6.0	6.7	3.0	10.9	7.5	3.2	1.5	51.4	37.0
6–10s	21	4.1	3.0	8.5	6.0	7.0	5.0	9.8	8.0	4.2	2.0	50.4	35.3
11+	30	3.9	1.8	8.2	5.0	4.3	3.0	5.4	4.0	3.9	2.0	37.0	29.3
Years of SR experience													
<1 year	16	2.9	2.5	8.8	3.5	5.4	3.5	10.2	4.8	3.9	1.4	46.6	24.6
1–3 years	52	3.7	3.0	8.5	6.0	5.9	3.0	10.5	7.3	3.7	2.0	49.2	36.5
4–6 years	25	4.2	2.0	7.9	5.0	5.4	3.0	8.0	6.0	4.4	2.3	43.9	34.0
7+ years	12	5.9	1.0	8.6	4.5	3.1	2.0	4.4	2.5	2.9	2.0	34.2	23.2

### Tasks and time spent

Table 2 reports the mean, median, and maximum durations of time spent completing core SR tasks. Due to extremely short durations reported for specific instructional tasks, we collapsed subcategories into an omnibus task category designated “instruction.” The cumulative duration across tasks averaged 30.7 hours (SD=30.0) with a median of 22 hours and a range of 2 to 219 hours.

**Table 2:** SR task time spent in hours

Task	Median duration	Average duration	Maximum duration
Core tasks			
Initial and follow-up interviews	2.0	3.9	50.0
Search strategy	5.0	8.4	100.0
Translating search	3.0	5.4	75.0
Documenting	2.0	3.0	20.0
Delivering	2.0	4.3	40.0
Writing	1.0	1.8	26.0
Related tasks			
Instruction	2.0	3.8	33.0
Additional tasks	0.0	2.2	35.0

### Additional tasks

The most frequently identified additional SR tasks were inclusion or exclusion criteria development; title, abstract, and article appraisal; and citation management or deduplication. Other additional tasks included compiling team minutes, using SR management software (e.g., DistillerSR and Covidence), retrieving full-text articles, searching reference lists, developing protocols, and selecting journals for publication.

### Relationship of experience level to task durations

In phase 2 of the analysis, we first examined relationships between level of experience and task duration. An additional 12 respondents’ data (11.4%) were excluded due to extreme outliers, leaving a total of 93 responses for this phase of the analysis. An exploratory PCA was performed to identify potential clusters of tasks and their relationships to level of librarian experience. VARIMAX rotation was performed on the results of the PCA, which included interview, search, translation, writing, and instruction as related to librarian experience level.

**Figure 2:**
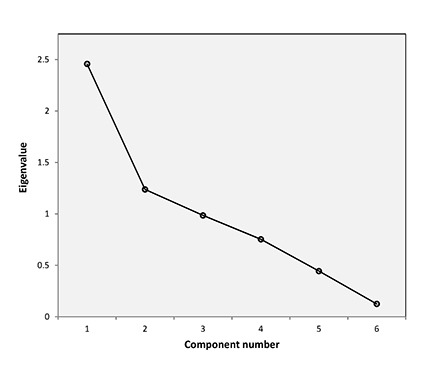
Scree plot

An examination of the scree plot of the PCA (Figure 2; data shown in Table 3) indicated that 2 components accounted for 61.6% of the variance. A scree plot helps visualize the relative importance of the components. The 2 components laying on the steep slope of the plot accounted for the most variance, and the remaining 4 contributed to smaller amounts of variance. The PCA matrix in Table 4 shows the 6 variables’ association with the first 2 discrete components: component 1 dubbed “Information processing” and component 2 dubbed “Interpersonal instruction/training.”

**Table 3:** Total variance explained

	Initial Eigenvalues			Extraction sums of squared loadings			Rotation sums of squared loadings		

	Total	% of variance	Cumulative %	Total	% of variance	Cumulative %	Total	% of variance	Cumulative %
1	2.457	40.951	40.951	2.457	40.951	40.951	2.382	39.697	39.697
2	1.237	20.618	61.569	1.237	20.618	61.569	1.312	21.872	61.569
3	0.985	16.419	77.988						
4	0.753	12.543	90.531						
5	0.444	7.4	97.93						
6	0.124	2.07	100.0						

**Table 4:** Principal components analysis (PCA) results

Variable	Component 1 loading (Information processing)	Component 2 loading (Interpersonal instruction/training)
Experience	−0.046	0.423
		
Interview	0.308	0.738
		
		
Search	0.910	−0.210
Translation	0.859	−0.336
Writing	0.801	0.051
		
		
Instruction	0.389	0.595

The analysis showed very close relationships (i.e., heavy loadings) for search, translation, and writing on the first component, which had almost no association with the experience level of the librarian. However, experience level was observed to load on the second component and, hence, was more closely related to interview and instruction than to the other variables. As seen in Table 4, the shaded areas in each component show the heavy loadings. Search, translation, and writing clustered together (0.910, 0.859, 0.801), and interview and instruction clustered together (0.738, 0.595).

VARIMAX rotation applied to these data converged in three iterations and essentially duplicated the PCA (Table 5). The experience level of the librarians was positively related to their interview and instructional task durations: more experienced librarians reported spending more time on the interview and instructional processes (e.g., interpersonal instruction), but their experience levels were unrelated to the amount of time spent on search, translation, and writing. These three tasks clustered in a way suggesting a relatively immutable cluster that was unaffected by the experience level of the librarians but determined perhaps by situational or project variables related to the intellectual and physical effort needed to conduct these information-processing operations. Hence, factors 1 and 2 in Table 5 mirror components 1 and 2 in Table 4, due to the similarity of their loadings (shown by the shaded cells in each component).”

**Table 5:** Factor structure after VARIMAX rotation

Variable	Factor 1 (Information processing)	Factor 2 (Interpersonal instruction/training)
Experience	−0.150	0.398
		
Interview	0.115	0.792
		
		
Search	0.934	0.022
Translation	0.916	−0.112
Writing	0.764	0.248
		
		
Instruction	0.229	0.673

### Resources searched

Respondents also reported how much of the search task time they spent searching resources by type. Literature databases accounted for 78.7% of total search time. Other resource types included pearling (6.3%), grey literature (5.9%), trial registries (4.4%), hand-searching (2.0%), and other (defined by respondents, 3.6%). It was interesting to note that librarians with 4 or more years of SR experience were more likely to report pearling references (65%), compared to librarians with fewer years of experience (50%).

### Qualitative findings

The survey encouraged comments from participants by providing comment boxes for each question set. This resulted in 130 text responses, adding a qualitative element to survey results and providing insight into other SR tasks that librarians perform. We classified the qualitative responses into the following thematic categories: experience, role of librarian, nature or complexity of SR, engagement level, and institutional expectations.

## DISCUSSION

As Allen and Olkin’s study predicted [14], our findings suggest there is no basis for establishing a standard time frame for librarian tasks in an SR. We suggest that the following areas are the major contributors to this variability: experience level, the librarian’s role, the nature and complexity of the SR, the level of engagement with other SR team members, and institutional expectations.

### Experience level

We identified potential trends in the aggregate data that appeared to link experience level, particularly greater number of SRs performed, with shorter times in the following tasks: instruction, interview, and translation of search queries to additional databases. However, a closer inspection of the data revealed that extreme outliers (i.e., librarians who reported seven or more years of experience) might have positively skewed these trends. When these data were removed from the analysis, there was no association between years of experience and search, translation, documentation, and writing tasks. However, weak positive correlations between years of experience and two of the tasks—providing instruction and conducting the interview—remained.

The PCA and exploratory FA with VARIMAX rotation reduced several variables down to two core dimensions. These results for the second dimension suggested that experienced librarians spent more time educating and interacting with faculty than junior librarians did. This finding is reasonable since senior librarians are likely to have performed more SRs and so understand the importance of defining the scope of the SR. They may also devote more time to clarifying the research question and have a better feel for collaborators’ experience levels. In contrast, time spent on more prosaic aspects (e.g., searching, translating, and writing) appears driven by other factors than experience level with SRs.

Based on our data, the overall pool of librarians who have conducted a large number of SRs is relatively small, and these SR “specialists” in our survey account for the lion’s share of efficiencies that can be attributed to experience level. With a median number of five SRs, it may simply be that most librarians do not yet have sufficient experience to reduce the amount of time that they spend on discrete tasks. Many librarians start their professional careers without the benefit of SR training in graduate school. While post-degree training courses are available, librarians may not have opportunities to engage in SRs as part of their regular duties, making it difficult to hone their SR skills [18].

### The librarian’s role

The reported levels of librarian involvement in SRs ranged from minimal consultation on search strategies to the role of principal investigator. This suggests that experiences vary, as is substantiated by the literature, which indicates that librarians working on SRs have two major roles: as expert searchers and as knowledge organizers [5, 13, 19]. Certainly, the librarian’s role in knowledge organization (i.e., data management) is central, and much of the literature addresses the time it takes to de-duplicate and document searches [20]. Librarians play a number of ancillary roles, such as peer-reviewing searches, writing or editing other portions of the manuscript, conducting statistical analyses, or acting as a consultant to the study team [5]; however, these activities are less common.

### Nature and complexity of systematic review topic

Several participants noted that the amount of time that they spent varies with the SR topic. The complexity of the subject, the librarian’s background knowledge of the topic, and the coauthor’s experience level all contributed to variation in time needed to develop an effective search strategy. The clearest finding was that SR search strategy development and translation took the largest proportion of librarians’ time. This corresponds with Gann and Pratt’s findings on the amount of time necessary to support an SR [16].

### Level of engagement with other investigators

Having a librarian as a coauthor or as a member of an SR team suggests better-reported search quality [4, 12]. Koffel and Rethlefsen found strong differences in the reporting of search elements among the disciplines of surgery, pediatrics, and cardiology; however, the disciplines most likely to include a librarian were also those most likely to include a reproducible search [20]. These findings are supported by qualitative comments in our study indicating that time spent in social interaction with other investigators (i.e., interview and instruction phases) appears to improve the quality of the search.

### Institutional expectations

A survey of supervisors at Canadian academic health sciences libraries showed a lack of consensus regarding the involvement of librarians in SRs. Supervisors most often expected librarians to engage in developing the search strategy, translating the search across databases, and managing citations [18]. However, institutional climate (supervisor or librarian point of view) might or might not affect the perception that participation in SRs should be part of a librarian’s core responsibilities [8, 10]. This literature corresponds to qualitative responses indicating ambivalence from some library administrations regarding the value of SR support and unrealistic expectations regarding how much time librarians should spend on SR tasks. Librarians also reported that they themselves had concerns over what, precisely, their responsibilities should be.

### Implications for library planning

Because the range of results shows a lack of consistent times for specific tasks, there is no prescriptive guideline to say how long an SR should take. This lack of specificity makes it challenging for administrators to plan for services and allocate staff time. It is also difficult for librarians who want to participate in an SR to anticipate the appropriate amount of time needed for specific tasks. Furthermore, librarians in academic settings often do not track the time they actually spent on SRs, nor are they required to track their time that closely. Librarians who did track their time tended to record only the type of search (SR) and estimated total time as an aggregate task, not the discrete elements of the SR.

Educating and training librarians to engage more fully in SRs poses a challenge. It is difficult to learn best practices or to adopt specific standards on one’s own, and formal training in conducting SRs may be cost and time prohibitive. However, for those librarians and libraries wishing to add SRs to their professional offerings, a full understanding of how to conduct and assess SRs for all staff members who are so engaged is critical for understanding the required staffing commitment and professional development.

### Limitations

One limitation of the survey was that we did not adequately discern the nature of the respondent’s role in the SR. Understanding whether a librarian was responsible for developing the search strategy or just served as a mentor or advisor could help remove one possible confounding influence that we observed in reporting task time. A second study limitation concerned the subjective appraisal of time based upon recall of the most recently conducted SR. If the most recent SR was a year ago, the librarians might or might not remember with sufficient clarity the time that they spent on those discrete tasks. A retrospective response bias for allocating similar amounts of time to each task would affect the relationships between time and task.

Future studies should also investigate the librarian’s experience in traditional reference and searching overall as well as any formal training that they may have had in conducting an SR, as opposed to solely SR experience. These factors would affect SR task time, such as search strategy development and documentation.

## CONCLUSIONS

Numerous studies have attempted to quantify the time librarians spend in supporting SRs [14–16]. Our research may be the first to report on specific, discrete SR-related tasks and to elucidate how much time individual practitioners take to complete them. For library managers to better understand future SR staffing needs, it is important for librarians to track their time spent on discrete SR tasks carefully and contemporaneously. This practice is likely to provide more accurate and useful data by preventing subjective recollection from distorting estimates of time spent on individual SR tasks.

While our findings did not produce prescriptive benchmarks for how long SR tasks should take, we believe that the findings of mean and median times will help administrators and librarians have a better idea of the time commitment required to engage in an SR. Prior to implementing an SR program, librarians need to thoroughly understand the breadth of tasks that could involve them. While experience may influence the time required for an SR, other factors identified in this study related to the nature of the research question under investigation may significantly affect the time required to conduct SRs.

## SUPPLEMENTAL FILE

Appendix Systematic review tasks survey instrument
