# Supplementary material for: It takes longer than you think: librarian time spent on systematic review tasks
Source: J Med Libr Assoc. 2018 Apr 1;106(2):198–207. doi: 10.5195/jmla.2018.323 (PMC5886502; doi:10.5195/jmla.2018.323)
Supplement: Appendix [file jmla-106-198-s001.pdf]

Journal of the Medical Library Association

Supplemental content to
J Med Libr Assoc. Apr;106(2):dx.doi.org/10.5195/jmla.2018.323
www.jmla.mlanet.org
© Bullers, Howard, Hanson, Kearns, Orriola, Polo, Sakmar 2018

It takes longer than you think: librarian time spent on systematic review tasks

Krystal Bullers, AHIP; Allison M. Howard, AHIP; Ardis Hanson; William D. Kearns; John J. Orriola; Randall L. Polo; Kristen A. Sakmar

APPENDIX

Systematic review tasks survey instrument

Q1. Have you participated in a systematic review (SR) in the last five years?
- O Yes
- O No

[Respondents answering "No" received the survey completion screen.]

Q2. **In total**, how many SRs have you worked on? Your best guess is fine. Count **ANY** SRs in which you have participated or are currently participating. It does not need to have been completed or published. _

Q3. How long have you participated in SRs in any capacity?
- O Less than a year
- O 1–3 years
- O 4–6 years
- O 7+ years

Q4. **In total**, how many SRs have you **COMPLETED**? Count any SR in which you have completed all the tasks assigned to you (i.e., you're done with your part.)____

[Subsequent questions asked participants about the most recent SR. If participant had completed 1 or more SRs, subsequent questions asked about their most recently completed SR. If participant reported that they had worked on 1 or more SR but had not (yet) completed their tasks, subsequent questions asked about their most recent SR.]

Section 1. Core librarian systematic review (SR) tasks

Q5. In your **MOST RECENTLY COMPLETED/MOST RECENT** SR, how much time did you spend on the following SR tasks **in HOURS**? If the time spent was less than an hour, please give a rough estimate of the portion of the hour. (For example, 0.25 for 15 minutes, 0.5 for 30 minutes, 0.75 for 45 minutes.)
_______ Conducting the initial interview and follow-up meetings
_______ Testing and developing search terms and strategy
_______ Translating search strategy to other databases
_______ Documenting search strategy (spreadsheet, word processing document, etc.)
_______ Delivering search results (bibliographic management tool, shared search statements, etc.)
_______ Writing search methodology section for manuscript (PRISMA, etc.)

JMLA Journal of the Medical Library Association

SC

Supplemental content to
J Med Libr Assoc. Apr;106(2):dx.doi.org/10.5195/jmla.2018.323
www.jmla.mlanet.org
© Bullers, Howard, Hanson, Kearns, Orriola, Polo, Sakmar 2018

Section 2. Tasks related to SR instruction

Q6. For this question, we want to explore the librarian's role in developing information skills critical to the successful SR. In your **MOST RECENTLY COMPLETED/MOST RECENT** SR, how much time did you spend on **INSTRUCTION in HOURS**? If the time spent was less than an hour, please give a rough estimate of the portion of the hour. (For example, 0.25 for 15 minutes, 0.5 for 30 minutes, 0.75 for 45 minutes.)

_______ Instruction in systematic review methodology (PICO, PRISMA, inclusion/exclusion criteria, etc.)
_______ Instruction in database management (MyNCBI, subject headings, etc.)
_______ Instruction in bibliographic management software (e.g., EndNote)
_______ Instruction in other (Please describe): ______________________________

Section 3. Other tasks not identified by the survey

Q7. In your **MOST RECENTLY COMPLETED/MOST RECENT** SR, did you perform any SR-related task not already covered, such as develop the protocol, create inclusion/exclusion criteria, or analyze data? Please report the time spent **in HOURS**. If the time spent was less than an hour, please give a rough estimate of the portion of the hour. (For example, 0.25 for 15 minutes, 0.5 for 30 minutes, 0.75 for 45 minutes.)

_______ Describe additional SR task: ______________________________
_______ Describe additional SR task: ______________________________
_______ Describe additional SR task: ______________________________

Q8. Comments (optional but welcome) ______________________________

Section 4. Time spent searching various types of resources

Q9. In your **MOST RECENTLY COMPLETED/MOST RECENT** SR, how much time did you spend searching the following **RESOURCE TYPES** as a percentage of your total time? (Your best guess is fine.) For this question, your choices **MUST total 100%**.

_______ Literature indexes/databases (MEDLINE, EMBASE, Web of Science, etc.)
_______ Pearling (searching the bibliographies of studies that you know you want to include)
_______ Trial registers (CCTR, ClinicalTrials.gov, etc.)
_______ Grey literature
_______ Hand searching (per Cochrane Handbook: "a manual page-by-page examination of the entire contents of a journal issue or conference proceedings to identify all eligible reports of trials")
_______ Other (please describe): ______________________________

Q10. Comments (optional but welcome): ______________________________

Q11. Is there anything we didn't ask you that you think we should know about librarians' time spent performing SRs? We welcome your thoughts and comments. ______________________________
